# Demand‐Resource Mismatch Explains Body Shrinkage in a Migratory Shorebird

**DOI:** 10.1111/gcb.70170

**Published:** 2025-04-16

**Authors:** Tim Oortwijn, Thomas K. Lameris, Mikhail K. Zhemchuzhnikov, Anne Dekinga, Job ten Horn, Dmitry Kutcherov, Simeon Lisovski, Theunis Piersma, Eldar Rakhimberdiev, Mikhail Y. Soloviev, Bernard Spaans, Evgeny E. Syroechkovsky, Pavel S. Tomkovich, Elena Zhemchuzhnikova, Jan A. van Gils

**Affiliations:** ^1^ Department of Coastal Systems NIOZ Royal Netherlands Institute for Sea Research Den Burg the Netherlands; ^2^ Conservation Ecology Group, Groningen Inst. For Evolutionary Life Sciences (GELIFES) University of Groningen Groningen the Netherlands; ^3^ Department of Biological Sciences University of Arkansas Fayetteville Arkansas USA; ^4^ AWI Alfred Wegener Institute Helmholtz Centre for Polar and Marine Research Potsdam Germany; ^5^ BirdEyes, Centre for Global Ecological Change at the Faculties of Science & Engineering and Campus Fryslân University of Groningen Leeuwarden the Netherlands; ^6^ Department of Theoretical and Computational Ecology, Institute for Biodiversity and Ecosystems Dynamics University of Amsterdam Amsterdam the Netherlands; ^7^ Zoological Museum and Department of Vertebrate Zoology Lomonosov Moscow State University Moscow Russia; ^8^ BirdsRussia Moscow Russia

**Keywords:** Arctic warming, body size, climate change, red knot, trophic mismatch

## Abstract

Recent observations of body size declines in animal populations have given rise to discussions of whether or not this is related to climate change‐induced temperature increases, with which the body size changes would follow Bergmann's rule. Although the debate is ongoing, the limited thermal benefits of currently observed size reductions make it unlikely that temperature increase shapes a direct selection pressure. Food constraints during early‐life development, which could be caused by mismatches between available resources and energetic demands, could cause smaller body sizes too. Here we investigate whether a decrease in body size, observed in a migratory shorebird, the red knot ( *Calidris canutus canutus* ) at their West‐African nonbreeding grounds over two decades, is linked to developmental plasticity during chick growth in the High Arctic. To do so, we combined datasets from both the wintering and breeding grounds on body size measurements (during chick growth and in fully grown juveniles), food availability, and diet inferred from stable isotopes deposited in feathers grown as chicks. From 2003 to 2021, stable‐isotope ratios revealed a decline in the dietary contribution of crane flies (Tipulidae, Diptera), the key food of growing chicks in the Arctic. On the breeding grounds, we observed that while the emergence of adult crane flies advanced along with earlier snowmelt dates, red knots did not adjust the timing of breeding, and this resulted in an increasing mismatch with the demands of growing chicks. As a result, chicks grew slower and, as observed on the wintering grounds, reached smaller final body sizes. Our results imply that increasing resource‐demand mismatches may lead to body shrinkage via plasticity during development. In this study, the increasing mismatch was linked with climate warming; the presented causal chain may explain other recent examples of body size reductions as well.

## Graphical abstract

Body size changes due to climate change can potentially be shaped by changing conditions during development. Juvenile red knots have smaller bodies in years with early snowmelt, caused by slower growth as chicks on the Arctic tundra, when they were mismatched with an earlier peak abundance of crane flies. Stable isotopes in juveniles' feathers, sampled on the West African wintering grounds but grown as chicks, revealed that the crane fly proportion in chick diets was lower in smaller juveniles and decreased over two decades of advancing snowmelt.

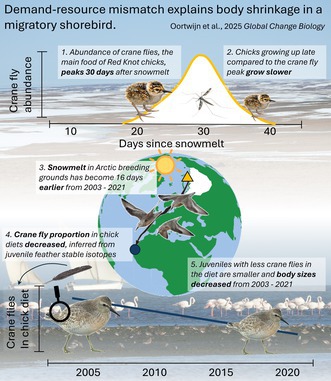

## 1 Introduction

Since Daufresne et al. (2009) suggested that declining body sizes could be a third universal response to global warming, the effect of climate change on animal body sizes is an often‐discussed topic. Reviews by Gardner et al. (2011) and by Sheridan and Bickford (2011) showed that body sizes are not always changing and that there were size reductions—but also increases. Bergmann's (1847) rule, the observation of animals tending to be larger at higher latitudes, often is presented as a potential explanation for body size changes in concert with increasing temperatures (Gardner et al. 2019; Goodman et al. 2012). However, the pattern recognized by Bergmann is not necessarily caused by latitudinal temperature gradients (Ashton 2002; Blackburn et al. 1999). Indeed, empirical studies that find declining body sizes often suggest that temperature is unlikely to be a direct cause (Husby et al. 2011; Salewski et al. 2014; Teplitsky and Millien 2014). Nord et al. (2024), for example, computed that body size reductions in birds observed by Youngflesh et al. (2022) are not providing substantial heat loss advantages consistent with the hypothesis to explain body shrinkage by selection for smaller individuals.

Alternatively, body size reductions are hypothesized to result from reduced growth during development. Although in most vertebrates growth is determinate and asymptotic, with variations thought to be mostly caused by genetic variation (Sebens 1987), several studies suggest that animal body size variation may result from plasticity in growth, or rather constraints in growth rate resulting from environmental factors (Dunlop and Morris 2018; Gebhardt‐Henrich and van Noordwijk 1994; Geist 1987; Richner 1989). Limited nutritional intake, in terms of food quality and quantity, is well known to lower growth rates (Hou et al. 2011; Richman et al. 2015; Wen et al. 2017). Nevertheless, if slower growth rates, and similarly slowed‐down maturation, lead to longer growth periods, animals would still be able to reach normal asymptotic body size (Schew and Ricklefs 1998). However, if maturation is not slowed down simultaneously with growth, tissues would lose their growth potential as they mature (Starck and Ricklefs 1998b), and growing animals reach smaller sizes. This may particularly hold for species or populations with short breeding seasons, for example migratory bird species, where offspring have a limited time to grow before they need to leave the breeding grounds at the end of summer.

Climate warming and other anthropogenic global changes are associated with biodiversity loss (WWF 2020), including worldwide insect declines (Wagner et al. 2021) and concerns for fish stock declines (Hilborn et al. 2020). Whereas reductions in resources for offspring may arise from reductions in parental provisioning to cope with higher temperatures (Cunningham et al. 2021), general resource declines will surely lead to reductions in the resources for offspring, such as the chicks of migratory birds (Bowler et al. 2019; Cury et al. 2011). Reductions in the available food for growing offspring may also stem from mismatches between the timing of maximum offspring growth and seasonal resource peaks (Cushing 1974). Mismatches are often considered to increase with climate warming (Visser 2008), although the generality of this consideration has been disputed (Kharouba and Wolkovich 2023; Zhemchuzhnikov et al. 2021). Demand‐resource mismatches have already been shown to reduce offspring growth rates as animals grow up in poor food conditions (Lameris et al. 2022), yet whether this may carry over to smaller mature body sizes remains to be shown.

Finding that juvenile red knots (measured during their southward migration to West Africa) have smaller body sizes in years with earlier snowmelt on the Arctic‐breeding grounds, van Gils et al. (2016) proposed that these smaller body sizes resulted from a demand‐resource mismatch during offspring growth, which is our study focus here. By examining juvenile red knots captured on their nonbreeding destination rather than in transit, we first confirm the link between (1) earlier snowmelt in the breeding grounds and (2) reduced asymptotic body size. To further examine the link with snowmelt, we analyzed whether (3) the timing of hatch relative to snowmelt affected the body size of chicks, measured on the tundra. To then study whether this relates to a potential demand‐resource mismatch, we (4) measured the timing of peak abundance of a key prey in relation to snowmelt and (5) analyzed whether the proportion of this prey in chick diets changed over two decades by measuring stable isotopes in the feathers of the juveniles captured on the nonbreeding grounds.

## 2 Material and Methods

### 2.1 Study Species and Locations

We studied the nominate subspecies of red knot *Calidris canutus canutus* , a medium‐sized shorebird (mean body mass of an adult in winter, 124 g, ten Horn et al. unpub. data). Red knots are a dimorphic species, with females being larger in size than males (Rooselaar 1983). They breed on the high‐Arctic tundra on the Taimyr Peninsula in Russia. Around mid‐June, females lay a four‐egg clutch, which is then incubated by both parents, after which chick care is provided by the male, with chicks fledging after two to three weeks (Loktionov et al. 2015). Birds then depart on southward migration, females departing mid‐July (after hatching) and males and juveniles departing between the end of July and late August (Piersma et al. 1992). They make a 9000 km migration to their West‐African wintering grounds, with the majority of birds overwintering in the intertidal ecosystem of Banc d'Arguin, Mauritania.

We studied breeding red knots on their breeding grounds at Knipovich Bay (76°04′ N, 98°32′ E) in the center of the breeding range of this population (Figure S1) during four summers (1990, 1991, 2018 and 2019), and on their wintering grounds in the Banc d'Arguin from 2003 to 2021 (El‐Hacen et al. 2024; Leyrer et al. 2012; van Gils et al. 2013).

### 2.2 Timing of Snowmelt in the Arctic Breeding Grounds

We used a satellite‐derived MODIS (MOD10A1.061) Terra Snow Cover Daily Global 500 m dataset (Hall et al. 2016) to estimate the date of snowmelt in spring on the entire breeding grounds of red knots (*canutus* subspecies, Figure S1) for the years 2003 to 2021. Masked daily Normalized‐Difference Snow Index (NDSI) from March until September using the MODIS water mask (MOD44W, 24‐02‐2000), cropped to the red knot breeding range (northern Taimyr Peninsula as defined by Lappo et al. (2012)), were downloaded (*n* = 3340) via the Google Earth Engine. For each pixel (*n* = 297,480), the valid NDSI values (scaled between 0 and 1) were first transformed into a binomial distribution with 0 < 0.4 >= 1 (established threshold for MODIS NDSI values (Sankey et al. 2015)). Next, an asymmetric Gaussian curve was fitted to the data of each year using a maximum likelihood approach. The time of snowmelt was then extracted as the day of the year in spring when the fitted curve fell below a 0.5 threshold, corresponding to 50% of the area being snow free.

We also used satellite data to estimate local snow cover at the study site in the Taimyr Peninsula for the years 1990–1991 and 2018–2019. As this time period spanned years from before the availability of higher‐resolution MODIS data (see above), we obtained lower‐resolution satellite data from two datasets compiled by the National Snow and Ice Data Centre (NSIDC), namely the weekly NH EASE‐Grid 2.0 Snow Cover and Sea Ice Extent (version 4, available from 1978–2017, 25 × 25 km grid) and IMS Northern Hemisphere Snow and Ice Analysis datasets (4 × 4 km grid, available from 2002–present). We obtained data for all grid cells in a radius of 35 km around the study site, which we used to calculate the date of snowmelt, defined as the first day with less than 50% snow cover. For validation, we also measured the date of snowmelt in a local grid in the vicinity of the study camp. Snow cover in the local grid was measured every 2 days from our arrival in the study area until the moment all snow had disappeared in a grid of 119 ha, using visual estimations for quadrats of 0.25 ha. Using linear interpolation, we calculated the day of 50% snowmelt as the first date at which snow cover in the grid was less than 50% (same method as described for the MODIS NDSI dataset). The date of snowmelt around the study camp correlated well with the date of snowmelt obtained from satellite data (Pearson's correlation: 0.97, *p* = 0.02, *n* = 4, Figure S2), but occurred on average 5.3 ± 2.6 (SD) days earlier. As the area surrounding the field camp was a low‐lying area with few nesting red knots, we continued to use satellite‐derived date of snowmelt.

### 2.3 Biometrics of Red Knots in the West‐African Wintering Grounds

We caught red knots around the village of Iwik, in Parc National du Banc d'Arguin (PNBA), Mauritania (20°14′ N, 16°06′ W) during winters (between November and April) from 2003 to 2021. The birds were usually caught with mist nets at night close to their high‐tide roosts, but sometimes cannon nets were used on the high‐tide roosts during the day (Leyrer et al. 2006). Age was determined from plumage characteristics (Prater et al. 1977). Bill length (measured from feather line to tip of bill) and tarsus length (measured from inter‐tarsal joint to the joint between tarsus and toes, with toes bent at 90°) were measured to the nearest 0.1 mm with calipers, and wing length (measured over folded and flattened wing, from bend of wing to tip of the 10th (longest) primary feather) was measured to the nearest mm with a ruler. For consistency of data, only five different persons measured the birds over this period, of which one (TP) being active the entire period. Measurements were also checked between observers. A blood sample was taken by pinching the brachial vein to later determine the sex using molecular marker techniques in the lab (van der Velde et al. 2017). To gather information on chick diets through stable isotopes in the feathers of juvenile individuals (that grow those feathers while being chicks on the Arctic tundra), the top half of a 6th primary covert was cut off and conserved in a small plastic bag or paper envelope.

### 2.4 Timing of Hatch and Chick Growth in the Arctic

#### 2.4.1 Hatch Dates

In June and July 1990–1991 and 2018–2019, we studied red knots at our study site in Taimyr Peninsula, which consists of Arctic tundra habitat with alternating valleys and hills, with red knots breeding mostly on the upper part of slopes.

In this study site we located nests during the laying and incubation period. We searched for nests by exploring suitable habitat for red knots on foot and flushing incubating adults from the nest, and by observing adult birds in the process of egg laying. Both males and females incubate the eggs, while it is usually the males that accompany the chicks after hatch (Tomkovich et al. 2018). In 2018 and 2019, in order to locate extra nests and broods, we lured adult males using territorial calls and captured them using an automated net, after which we equipped males with radio transmitters (Holohil BD‐2, 1.4 g). Thereafter we located nests by tracking males during incubation. For nests found without the help of radio‐tagged males, we trapped males on the nest and attached radio transmitters during mid‐incubation.

We predicted hatch dates from the time required to complete the clutch to four eggs for clutches detected during laying and by using the flotation method for clutches detected during incubation (Liebezeit et al. 2007). Nests were visited shortly prior to the predicted hatch dates and subsequently revisited every two days. Hatch date was defined as the day chicks were breaking out of their eggs or were laying in the nest with wet downy feathers. When we encountered chicks with fluffy downy feathers in the nest cup, we defined the day prior to the day of visit as the observed hatch date. It often occurred that we caught a brood that had hatched in an undetected nest. In this case, the hatch date was estimated from the size of the 10th primary of chicks in a brood (see below). The number of nests and broods found using these various methods is shown in Table S1. We used a combination of observed and estimated hatch dates to calculate average hatch dates per year.

#### 2.4.2 Chick Biometrics

After the nest phase, we traversed study areas on foot to search for chicks, which leave the nest cup a day after hatch and are then accompanied by the male parent. In 2018 and 2019, we located broods by tracking radio‐tagged males, as well as by playing recordings of chick distress calls, attracting adult males that could then lead us to their brood. Chicks were captured by hand or mist net. In case we encountered new broods with an untagged adult, this adult male was captured by using mist nets in combination with chick distress calls and equipped with a radio tag.

Each chick was banded with a metal band engraved with a unique code upon its first encounter, which was shortly after hatch for known nests or (usually) later for broods from unknown nests. We measured the length of the bill, the tarsus (see above) and the 10th primary (measured where the shaft is attached to the skin until the tip of the feather) to the nearest 0.1 mm using calipers. Chicks were weighed to the nearest 0.1 g using a scale. In 2018 and 2019, a small blood sample was taken from the leg vein of each chick and stored on an FTA card. The sex of chicks was determined using molecular marker techniques (van der Velde et al. 2017).

Age of chicks with unknown hatching dates was estimated using the correlation between age and the length of the 10th primary. We assume that 10th primary length is an adequate proxy for age as it shows limited variation between years and thus appears relatively uninfluenced by food availability (described in Lameris et al. (2022)). In case chicks were encountered (and measured) multiple times, we used the length of the 10th primary as measured during the first encounter. Estimated age was averaged per brood, as we assumed chicks in one brood to have all hatched on the same date.

#### 2.4.3 Temperature Data

For our study site in Taimyr Peninsula we downloaded modelled air temperature at 2 m above surface at six‐hour resolution for the period 1 June to 30 August for the years 1990–2019 from the NCEP reanalysis numerical weather model (spatial resolution 1.875° × 1.875° gaussian grid, Kalnay et al. (1996), using the R package “RNCEP,” Kemp et al. (2012)). Modelled temperature was strongly correlated with temperature measured by a weather station at the study site in 2018 and 2019 (Lameris et al. 2022).

### 2.5 Chick Diet, Resource Abundance and Demand‐Resource Mismatch

#### 2.5.1 Arthropod Abundance

In 2018 and 2019 we collected data on arthropod availability in a series of 45 yellow round (⌀ = 9 cm) pitfall trap stations placed in grids and transects in different locations in the study area, as described in detail in Zhemchuzhnikov, Lameris, et al. (2024). Stations varied in the thickness of the snow layer, the elevation, and the amount of sunlight that they received. Hence, there was strong variation in the local, station‐specific date at which a station was snow free. When the local snow layer was close to breaking open, we made daily visits to stations to record the day of snow disappearance. This allowed us to determine snow free dates for 13 traps in 2018 and 32 in 2019. After stations were free from snow, pitfall traps were installed and filled with propylene glycol to trap arthropods. At 5 stations we collected the trapped arthropods every day, while at the other 40 stations the samples were collected every 5 days. 25 stations were sampled in both years, and 20 stations were only sampled in 2019 (all of which were 5‐day interval stations). All trapped arthropods were stored in ethanol and taken to the laboratory after the field season, except for collembola, because the trapping method was not suitable to determine abundance for this subclass.

Captured arthropods were identified up to family level, and length was measured to the nearest mm. Earthworms and butterflies were excluded, as these were not considered potential prey for chicks. We determined biomass dry mass in mg of individual arthropods by using family‐specific length‐biomass relationships measured at our study site (Versluijs et al. 2023). For several families, we did not measure these relationships at our study site and used length‐biomass relationships measured at another Arctic site (Versluijs et al. 2023) (Zackenberg, Greenland; for families Anthomyiidae, Scathophagidae and Syrphidae, totaling 1.3% of the data) or order‐specific relationships (Hódar 1997; Sample et al. 1993) for 5 families, totaling < 1% of the data.

#### 2.5.2 Chick Diet

To enable long‐term diet analysis from stable isotopes in the feathers of juveniles, sampled in Mauritania but grown on the Arctic tundra, prior knowledge on chick diets is essential. This was gained from chick feces sampled in Taimyr. During the capture and measurements, “waiting” chicks were held in a canvas bag until release. After release, we collected chick feces from this bag and stored these in Eppendorf tubes with ethanol. In total we collected 69 fecal samples from 19 broods in 2018 and 20 broods in 2019.

We used metabarcoding methods to determine chick diet from feces following the methods described in Verkuil et al. (2022) and Zhemchuzhnikov, Zhemchuzhnikova, et al. (2024) with respect to DNA extraction, PCR protocol with primers on the CO‐I gene, and settings for the bioinformatics workflow based on OTU clustering with a 97% identity cut‐off. Taxonomy assignment was done based on a custom database containing 69 newly derived Sanger sequences of all insect morphotypes caught in the pitfall traps at the study site in Taimyr plus 1337 sequences of Arctic insects taken from GenBank. A detailed description of the molecular genetic methods and the pipeline for processing the data as well as the reference database are provided in (Zhemchuzhnikov, Zhemchuzhnikova, et al. 2024). Using these methods, we extracted the number of barcoding reads per arthropod family (except for the Araneae, Collembola and Acari) and used the relative number of reads as a fraction in the diet. As for some broods, multiple samples were collected for a single observation moment, and some samples were analyzed multiple times, we averaged diet fractions per brood and per observation date, resulting in a total of 52 samples.

We considered all arthropod families that on average contributed more than 1% to the diet as key prey families, and those were used for the stable‐isotope analyses. To determine stable‐isotope ratios of those families, individuals were selected from the pitfall samples of 2019 (equally distributed over the season).

For chicks with growing body feathers, we collected 2–3 feathers for stable‐isotope analysis, besides fecal samples. While fecal samples provide snapshots of the diet and provide essential information to enable stable‐isotope analyses, stable isotopes accumulated in those feathers contain information on the diet over a longer period and are used to link diet to relative hatch date. The analysis is the same as for extracting diet information from juvenile feathers collected in Mauritania.

#### 2.5.3 Determining Stable Isotope Values

Insect samples were freeze dried prior to isotope analysis. Feather samples were first rinsed in ethanol, then in hexane, in order to remove wax and dirt, and airdried afterwards. All samples were weighed into tin capsules (0.5–1 mg). For small insects, this meant that several individuals together were measured to reach the lower mass limit, while big insects (heavier than the upper mass limit) were measured in pieces which were averaged afterwards. Feather samples were cut from the tip until the right amount was reached. Tin cups were loaded on a Flash 2000 EA with a MAS 200 autosampler, connected via a CONFLO IV to a DELTA V ADVANTAGE irMS (Thermo Scientific, Bremen, Germany). Certified standards used to determine the nitrogen isotopic compositions were Acetanilide, Casein, and Urea. The values are expressed in *δ* (‰) notation relative to air.

### 2.6 Statistical Analyses

We used a combination of generalized linear models (GLMs), generalized linear mixed models (GLMMs) and a structural equations model (SEM) to test relationships between variables of interest in R 4.2.1 (R Core Team 2022). Details on GLMs and GLMMs are given here, while details on the SEM are given at the end of this section.

We constructed GLMs and GLMMs including all combinations of predictor variables of interest and compared model performance using Akaike's information criterion corrected for small sample sizes (AICc, Burnham and Anderson 2004). The model with the lowest AICc was chosen as our best model. Models within 2 ΔAICc of the best model were considered competitive if these did not contain extra, potentially uninformative parameters compared to the final model (Arnold 2010), and in these cases we used model averaging of the remaining competitive models. Specifics for each model used for the different analyses are described in the relevant sections below.

#### 2.6.1 Date of Snowmelt on the Arctic Breeding Grounds

For our first objective, to confirm the trend of earlier snowmelt on the breeding grounds, we tested whether the mean date of snowmelt (using the MODIS snow cover data of the entire breeding range) changed over the years using GLMs, including year as an independent variable (Table S2).

#### 2.6.2 Biometrics of Red Knots in the West‐African Wintering Grounds

For our second objective, to confirm a trend in body size of red knots measured in West Africa wintering grounds, the first principal component of the three measured structural sizes bill, tarsus, and wing length (scaled by unit variance) was used as a measure for body size. While being aware that this combined measure might not be valuable if the morphological traits change in opposite directions, it is coherent with the earlier analysis by van Gils et al. (2016), where all traits decreased. To examine juvenile body size changes over time, the variation in body size (PC1) and structural sizes was analyzed by comparing GLMs including year and sex, and their interaction as fixed effects. For visual purposes as well as comparative purposes, we also calculated changes in juvenile body size as a percentage, for which details can be found in the Supporting Information. For the main analysis, PCA was performed on juveniles only. To compare body size changes of juveniles with those of adults, a PCA was performed on their data combined.

#### 2.6.3 Timing of Hatch and Chick Growth in the Arctic

For our third objective, to test if the timing of hatch relative to snowmelt affected the body size of chicks measured on the tundra, we analyzed changes in chick hatch dates and chick growth rate. We analyzed whether brood‐specific hatch dates changed over the years using GLMs, including year as an independent variable. The model including both observed and estimated hatch dates yielded the same result as a model including observed hatch dates only (no effect of year on hatch dates, Table S2).

We estimated chick growth at the population‐level and for all years combined using cross‐individual data in growth models, from which we extracted residuals to further analyze the relationship with relative hatch date and other environmental variables. We chose this approach rather than including environmental variables in growth models, as such fixed effects need to be included in connection to multiple model parameters (inflexion point *t* and growth rate *k*), resulting in overly complex models, especially when also considering random effects of individuals.

Growth models of bill length, tarsus length, and their combined first principal component were fitted on data from individuals with known age together with individuals with predicted age. We used von Bertalanffy growth models to model bill length and the first principal component and logistic growth models to model tarsus length and body mass, as these performed best in a comparison of different growth models (Table S3). As chicks usually have not yet reached adult structural size at fledging, allowing models to estimate asymptotes for such measures may result in unrealistically depressed growth curves when older chicks are relatively underrepresented (Tjørve and Tjørve 2010). We therefore set a fixed upper asymptote *A* for models of bill length and tarsus length using the mean structural size of juvenile birds as measured on the wintering grounds (using the mean of the male‐ and female‐specific averages, to correct for unequal sample size between sexes). We calculated the asymptote for the first principal component by including mean bill length, tarsus length data, and body mass of juveniles in the principal component analyses and using its first principal component value. We chose not to set a fixed asymptote for body mass growth models as body mass varies throughout the year and values from the wintering grounds may not be representative.

Chicks were often captured more than once, and therefore, we included chick identity as a random intercept on growth‐rate parameter *k*. We estimated model parameters (growth‐rate *k* and horizontal placement of inflexion point *T*, asymptote *A* only for body mass growth models) from nonlinear least squares (Figure S3, Table S4), using the R package “nlme” (Pinheiro et al. 2017). For each individual chick, we calculated a “chick condition index,” by dividing the residuals from the growth models by the structural size (bill length, tarsus length, body mass or principal component value) at that age predicted from the same model.

For each chick, we calculated (i) a relative hatch date (RHD) as the individual hatch date minus the date of (local) snowmelt for that year, and (ii) the average temperature during the 3 days preceding catch from the RNCEP temperature data. We then analyzed the effects of these variables on chick condition index (for bill length, tarsus length, body mass and the first principal component value, see above) using GLMMs, where we included relative hatch date and average temperature as fixed effects and year and chick identity as random intercepts. In these analyses, we excluded chicks younger than 2 days old, as up to 2 days after hatching, chicks mostly survive on their yolk sacks (Starck and Ricklefs 1998a) and variation in condition up to this age is unlikely to be related to the availability of food. Each analysis was conducted for chicks of known and predicted age combined, as well as for chicks of known age only. As we found that the best performing model never differed between these different datasets, we only report on the models including both chicks of known and predicted age combined in the results. We tested whether the inclusion of sex as a random effect in growth models affected the relationship between chick condition index and relative hatch date but found no significant effect and thus did not further include sex in our growth models. For visualization, we also expressed chick body size as a percentage relative to the size at the earliest relative hatch date. Details on analyses including sex and on the calculation of body size as a percentage can be found in the Supporting Information.

#### 2.6.4 Chick Diet, Resource Abundance and Demand‐Resource Mismatch

For our fourth objective, to study the potential demand‐resource mismatch by measuring the timing of peak abundance of a key prey in relation to snowmelt, we first identified key prey of red knot chicks, followed by analyzing their timing of peak abundance.

We determined whether red knot chicks selected for key prey families by calculating the Ivlev selection index, thereby relating chick diet to the availability of arthropods as measured from pitfalls. For this analysis, we used data from a grid consisting of 10 pitfall traps located in an area where we found nests and broods of red knots in 2018 and 2019. We totaled biomass per family for each 5‐day sampling period. From these data we computed daily biomass values per family using linear interpolation, where we corrected for the average sampling date (emptying date‐2.5 days) and for the sampling interval by dividing the total value by 5. We also calculated per family the proportion of biomass available per day (relative to the biomass of all arthropods combined). For each key prey family (as determined from diet analyses, see above) in each dropping sample, we calculated the Ivlev index based on the observed proportion (O) of a certain family in the dropping sample and the expected proportion (E) of biomass found in the pitfalls on the sampling date:

$$\text{Ivlev index}=\frac{O-E}{O+E}$$

All values thus range between ‐1 and 1, with values above 0 indicating preference and values less than 0 indicating avoidance. Results for all key prey families can be found in Table S5.

The results of the metabarcoding analysis and above analysis showed that crane flies (*Tipulidae*) are the most consumed prey (see results). To further analyze the timing of crane fly peak abundance, we counted for each of the 45 pitfall stations the number of crane flies trapped per day and linked this to the number of days after the local, station‐specific snow free date. To summarize these data graphically, we binned the number of days after snowmelt to 5‐day intervals, starting at 0. This also enabled us to calculate, per station, the average number of days after snowmelt at which a crane fly was trapped. We tested whether this metric differed between years with a t‐test.

#### 2.6.5 The Link Between Chick Diet, Growth Rate and Body Size

For our final objective, to study if the proportion of key prey in chick diets changed over two decades, we first estimated the diet composition from the stable nitrogen isotopes in the feathers of the juvenile red knots (sampled in Mauritania 2003–2021). We used simmr (Parnell 2019), an R package designed for stable‐isotope mixing models, where we included the seven insect families that formed more than 1% of chick diets, based on DNA barcoding results (Table S5) as potential food sources. We used discrimination factors (± SD) specific for red knot feathers, 3.53 ± 0.30 for the primary coverts of juvenile birds and 3.33 ± 0.28 for the body feathers of the chicks (Oortwijn et al. 2023). The model works with a Markov chain Monte Carlo function that runs four chains with 10,000 iterations each, of which the first 1000 are dropped. We ran the model for each sample and extracted the mean (for further analyses) and standard deviation (only for visualization) of the estimated crane fly proportion in the diet.

GLMs were used to determine whether variation in the proportion of crane flies in the diet was affected by the timing of hatch (for data from sampled chicks in the breeding grounds) and by the date of snowmelt (for data from sampled juveniles in the wintering grounds). For data from chicks, we included relative hatch date and year as fixed effects and brood ID as a random intercept. For data from juveniles, models included fixed effects of year, snowmelt date, and sex (and their interactions).

To estimate the statistical causality among the observed phenological variables, we used path analysis, a special case of the structural equations modeling framework (Rakhimberdiev et al. 2018). In the proposed model, we estimated the strengths of the potential causal relationships between the date of snowmelt, the proportion of crane flies in the chick diet, and body size. The model contained two independent variables, time (year) and sex (male | female). Variables were assumed to have latent states and variable‐specific normally distributed errors.

1. ${\text{Date of Snowmelt}}_{i}=b_{2.2}+b_{1.2}\times \mathrm{Tim}e_{i}+\epsilon_{i};\epsilon_{i}\in \text{Norm}\left(0,\sigma_{\text{snowmelt}}^{2}\right)$
2. $\begin{array}{c} {\text{Proportion of crane flies in diet}}_{i}=b_{3.3}+b_{1.3}\times \mathrm{Tim}e_{i}+b_{2.3}\times \end{array}$ $\begin{array}{c} {\text{Date of Snowmelt}}_{i}+\epsilon_{i};\epsilon_{i}\in \text{Norm}\left(0,\sigma_{\text{proportion of crane flies in diet}}^{2}\right) \end{array}$
3. $\begin{array}{c} {\text{Body size}}_{i}=b_{4.4}+b_{1.4}\times \mathrm{Tim}e_{i}+b_{2.4}\times {\text{Date of Snowmelt}}_{i}+b_{3.4}\times \end{array}$ $\begin{array}{c} {\text{Proportion of crane flies in diet}}_{i}+b_{4.1}\times \mathrm{Se}x_{i}+\epsilon_{i};\epsilon_{i}\in \text{Norm}\left(0,\sigma_{\text{Body size}}^{2}\right) \end{array}$

Date of snowmelt on Taimyr Peninsula was hypothesized to affect the proportion of crane flies in the diet, and the proportion of crane flies in the diet to affect body size. In addition, the sex of individual birds was also hypothesized to affect body size. We used mean estimates for date of snowmelt without uncertainties to ease the combination with other data. All the variables were centered to have zero mean and scaled. Effects of variables were estimated as maximum probability of parameter to be strictly positive or strictly negative. The model parameters were estimated with MCMC JAGS sampler (Plummer 2003) using the R package “jagsUI” (Kellner and Meredith 2021). To test the reliability of parameter estimates, we simulated a new dataset of the size of our data with the modelled parameter estimates and parameterized a new model on the simulated data. Since we found no biases in model estimates based on simulated data, we conclude that the original model was reliable.

## 3 Results

### 3.1 Timing of Snowmelt in the Arctic Breeding Grounds

**FIGURE 1:**
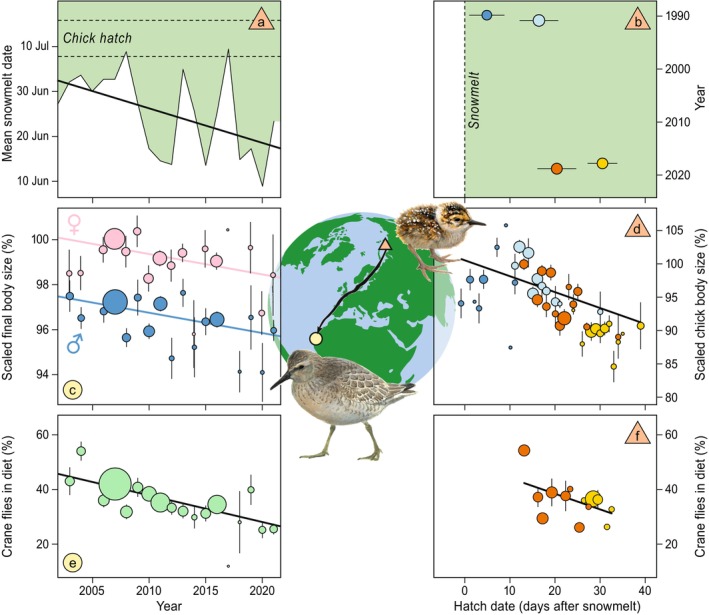
Earlier snowmelt dates result in larger demand‐resource mismatch, causing a decline in growth and final body size of juvenile red knots, evident from fewer crane flies in their tundra diet. (a) Mean snowmelt dates in the entire breeding areas on the Taimyr Peninsula have advanced over 19 years (solid thick line shows fitted GLM), while mean hatch dates did not change (dotted lines indicate SDs from mean the hatch date). The white‐green transition indicates the period before (white) and after snowmelt (green). (b) Chicks therefore hatch later relative to local snowmelt dates at the Arctic study site in more recent years, means ± SD of hatch dates are shown for 93 broods. Years (1990, 1991, 2018 and 2019) have different colors like in (d) and (f). (c) Final body size (based on tarsus, bill and wing length) of juvenile birds measured at the Mauritanian wintering grounds (West Africa), decreased over those 19 years of advancing snowmelt dates. Body size is scaled to a 100% reference value (predicted size of female juvenile hatched in 2003). Means ± SE of scaled body size are shown per winter (year in which the winter started) and per sex (males (*N* = 274) in blue, females (*N* = 318) in pink). Size of dots scales with sample size. Lines show fitted GLMs relating body size to (hatch)year per sex (and colored by sex). (d) Chick body size (based on bill length, tarsus length and body mass) decreases with hatch date (in days after snowmelt). Body size is scaled to a 100% reference value (predicted size of chick hatched at the earliest observed hatch date, one day before 50% snowmelt). Lines show fitted GLMMs per year. Means ± SE are shown per hatch date, dot size scales with sample size (total 262 measurements on 208 chicks) and colors correspond to different years (see b). (e) Over years, the proportion of crane flies in the diet of growing chicks has decreased, as estimated from *δ* ^15^N isotope ratios in the feathers of 592 juveniles (grown as chicks on the tundra) caught on the wintering grounds in Mauritania. Means ± SE are shown per year, dot size scales with sample size and line shows fitted GLM. (f) Later hatched chicks, relative to snowmelt, had lower proportions of crane flies in their diet. Estimates are based on *δ* ^15^N isotopes ratios from feather samples of 37 chicks. Line shows GLMM‐fit across both years. Means ± SE are shown per hatch date, dot size indicates samples size and colors correspond to different years (see b).

**FIGURE 2:**
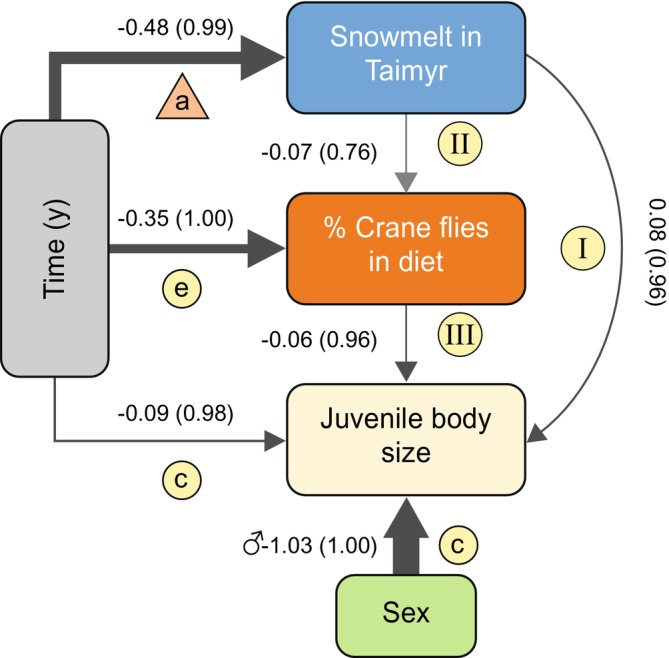
Schematic representation of the structural equations model. Arrows indicate direction of causal relationships between the variables, with arrow width proportional to scaled coefficient strength and arrow colour hue proportional to probabilities. Next to the arrows the coefficients are given with probabilities P(|λ| > 0) between brackets. Other information on coefficients uncertainty is given in Supporting Information Table S6. The variable Time represents linear temporal trend (measured over years), the variable Sex represents male or female sex of individual birds. Letters a, c and e correspond to plots in Figure 1 and letters I, II and III correspond to plots in Figure S4.

In the breeding grounds of red knots in high‐Arctic Siberia, mean snowmelt dates in summer have advanced rapidly between 2003 and 2021 by −0.87 ± 0.33 (SD) days per year (Figure 1a, probability P(|λ| > 0) from structural equation model = 0.99, Figure 2, Tables S2 and S6).

### 3.2 Biometrics of Red Knots in West Africa

On the main wintering grounds of red knots in Banc d'Arguin, Mauritania, body sizes of juvenile red knots decreased over the last two decades, with an annual change of −0.04 ± 0.01 mm (SE) in bill length, −0.04 ± 0.01 mm in tarsus length, and −0.10 ± 0.03 mm in wing length (first principal component of these three measures (explaining 56.7% of the variation): −0.05 ± 0.01, P(|λ| > 0) = 0.98, phenotypic SDs: −0.04, Figures 1c and 2, Tables S6 and S7). This means that juveniles from 2021 were on average 1.7% smaller than juveniles from 2003 (based on PC1, Figure 1c, for tarsus, bill and wing this is, respectively, 2.1%, 1.9% and 1.1% smaller). The body size of juveniles was smaller after breeding seasons with an early snowmelt in the Arctic (per earlier snowmelt day, tarsus length ± SE: −0.009 ± 0.006 mm, bill length ± SE: −0.03 ± 0.01 mm, wing length ± SE: −0.06 ± 0.02 mm, PC1 = −0.02 ± 0.006, P(|λ| > 0) = 0.96, Figures 2 and S4I, Tables S6 and S7).

### 3.3 Timing of Hatch and Chick Growth in the Arctic

Despite an advancing date of snowmelt on their breeding grounds, red knots did not advance the timing of breeding (comparing 1990, 1991, 2018, 2019), nor did they adjust to changing snowmelt dates (average hatch date ± SD: July 12th ± 4 days, Table S2). This means that, relative to snowmelt date, chicks hatched later in recent years (Figure 1b).

Chicks hatching later after snowmelt tended to grow slower (relative hatch date was present in best models explaining chick condition index, Table S8), also when controlling for temperature variation. In comparison to chicks hatching at the date of snowmelt in 1990, for every day that hatching occurred later, chicks showed a reduction of 0.24% ± 0.05% in body size (Figure 1d). For tarsus length and body mass, a reduction was present only for chicks hatching more than 10 days after snowmelt, after which chicks showed a reduction of 0.35% ± 0.23% for tarsus length and 0.53% ± 0.23% for body mass for every day of later hatch (Figure S5, Table S8). For bill length, no reduction in size occurred within years, although chicks hatching in years with on average later hatch since snowmelt had shorter bills (Figure S5, Table S8).

### 3.4 Chick Diet, Resource Abundance and Demand‐Resource Mismatch

**FIGURE 3:**
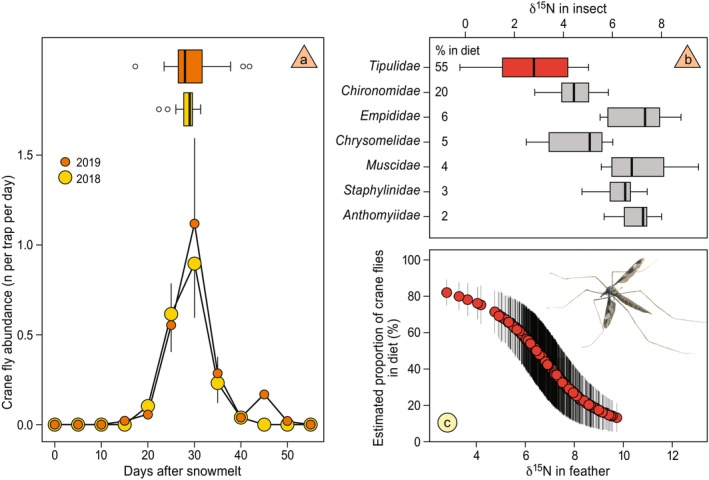
Crane fly abundance peaks four weeks after snowmelt and their distinct stable nitrogen value allows estimation of their proportion in red knot diets. (a) Crane fly abundance as the number per pitfall trap per day over time (means ± SE), where date of snowmelt is pitfall‐specific. Boxplots in the top of the graph show the average day on which crane flies were caught for each pitfall trap for both years. (b) Variation in *δ* ^15^N of the most important prey of red knot chicks (based on DNA barcoding). (c) Proportions of crane flies in the diet of red knot chicks, estimated by stable‐isotope mixing model on *δ* ^15^N values in their feathers collected in juvenile stage (means ± SD of model output). Note that the position of the *δ* ^15^N feather axis is corrected for the discrimination factor (3.53), such that horizontal axes in (b) and (c) align.

DNA barcoding of feces of chicks showed that crane flies formed the main part of the diet (mean ± SE per fecal sample: 54.8% ± 5.9%, *n* = 52) and that chicks actively select for this prey (average Ivlev index: 0.1, values > 0.0 indicate preference, Table S5). Another substantial part of the diet consisted of non‐biting midges of the family Chironomidae (mean ± SE: 20.1% ± 5.5%). Other insect families contributed little to the total diet (6% or less per family, Figure 3b) and were rarely selected for (Table S5). Crane fly abundance peaked 29.0 ± 4.9 days after the date of snowmelt (date of snowmelt determined per pitfall (2018: *N* = 13, 2019: *N* = 32), no difference in peak timing between years, *t*_13,32_ = −0.89, *p* = 0.38, Figure 3a). Numbers of crane flies rapidly dwindled after the peak (Figure 3a), and it is thus likely that fewer crane flies were available to growing red knot chicks in years with early snowmelt.

### 3.5 The Link Between Chick Diet, Growth Rate and Body Size

The *δ* ^15^N value of crane flies was lower than that of the other consumed arthropod families (Figure 2b, Table S5), which allowed us to apply a stable‐isotope mixing model to estimate the proportion of crane flies in the diet of individual chicks (Figure 2c). Over the period 2003–2021, the proportion of crane flies in the diet of birds captured in West Africa decreased (slope ± SE: −0.97 ± 0.21 percentage‐point per year, P(|λ| > 0) = 1.00, Figures 1e and 2, Tables S6 and S7) and growing chicks that had consumed proportionally fewer crane flies were smaller when measured upon arrival in West Africa (per % lower proportion of crane flies in the diet, tarsus length: −0.01 ± 0.004 mm, bill length: −0.005 ± 0.005 mm, wing length: −0.02 ± 0.01 mm, PC1: −0.01 ± 0.003 P(|λ| > 0) = 0.96, Figures 2 and S4III, Table S6). The proportion of crane flies in the diet tended to be lower in years with an earlier snowmelt (−0.29 ± 0.07 percentage‐point per earlier snowmelt day, Figure S4II), although this probability was relatively low (P(|λ| > 0) = 0.76, Figure 2, Table S6). Another indication of a mismatch comes from the proportion of crane flies in the diet estimated from feathers of growing chicks on the tundra: chicks hatching later after snowmelt had a lower proportion of crane flies in their diet (−0.6 ± 0.3 percentage‐point per later day of hatch, Figure 1f, although intercept‐only model was competitive, Table S9).

## 4 Discussion

Using data from the wintering grounds of red knots, we confirm a link between smaller body sizes of juveniles measured after arrival at their wintering destination and earlier dates of snowmelt on their Arctic‐breeding grounds, 9000 km away along a great circle route. This also became visible as a trend across years. Data from the breeding grounds further suggest that chicks are hatching progressively later after snowmelt, and that this is associated with slower growth. With late‐hatching chicks growing up after the seasonal peak in the abundance of crane flies, slow growth appears to be caused by a demand‐resource mismatch. Stable‐isotope signals in grown feathers of juveniles, reflecting the diet of chicks during growth, showed a positive correlation between the contribution of crane flies and body size. This seems strong evidence that earlier snowmelt in the Arctic is causing demand‐resource mismatches leading to slower chick growth.

### 4.1 Demand‐Resource Mismatches

Mismatches between seasonal resource peaks and chick demands have been shown extensively in temperate systems (Visser and Both 2005; Zhemchuzhnikov et al. 2021), but also for Arctic‐breeding shorebirds (Lameris et al. 2022; McKinnon et al. 2012; Saalfeld et al. 2019). Whether or not such mismatches are occurring more and more will depend on the rate of change in the phenology of both the food sources and the birds. Arthropod peaks in the Arctic depend mostly on snowmelt dates and temperatures (Chagnon‐Lafortune et al. 2024), and because trends in those variables differ between Arctic sites (Box et al. 2019; Taylor et al. 2022), phenological shifts are also not consistently found across all sites (Kwon et al. 2019; Schmidt et al. 2023). In addition, such phenological changes will differ between arthropod taxa (Koltz et al. 2018). Focusing on a single taxon in a single site may show clearer phenological patterns, such as the clear peaks in abundance of crane flies 29 days after snowmelt found in our study, even though only data from two years were available. Emergence of crane flies has earlier been shown to be strongly linked to the timing of snowmelt (MacLean Jr. and Pitelka 1971; Rakhimberdiev et al. 2018) and advancing crane fly emergence with earlier snowmelt dates has been previously recorded (Rakhimberdiev et al. 2018). Concerning shorebird laying dates, some shorebird species have been shown to adjust laying dates strongly to the timing of snowmelt (Meltofte et al. 2021; Ruthrauff et al. 2021), which would allow closer matching to the timing of food peaks (Kwon et al. 2019). By showing relatively little variation in egg‐laying dates under changing timing of snowmelt at various places in the Arctic (Lameris et al. 2022), red knots may be somewhat exceptional but would make their chicks more susceptible to mismatches.

We found the crane fly diet fraction to decrease over time, rather than only being linked to variation in the date of snowmelt. This suggests that besides an advancement of the emergence of crane fly imagos and thus their availability to red knot chicks, a second mechanism may explain the observed decline of crane flies in the diet of chicks over time. Arctic arthropod species show changes in both abundance and body size in response to a warming climate (e.g., Botsch et al. 2024; Daly et al. 2024), which may be related to conditions experienced by larvae in the soil (Høye et al. 2021). The rapid warming in north‐central Russia, both during summer and winter (Box et al. 2019; Rantanen et al. 2022), could negatively impact crane fly abundance and size at the imago stage through changes in the larval phase, especially considering that their larvae live in the soil for several years before emergence (MacLean 1973). Although red knot chicks may switch to feed on different arthropods (Zhemchuzhnikov, Zhemchuzhnikova, et al. 2024), these are far less profitable. Crane flies are easy to catch when crawling over the tundra and are of relatively large size (mean mass ± SD: 6.56 ± 2.06 mg, data from Versluijs et al. (2023)). For comparison, a red knot chick will need to consume about 66 non‐biting midges (mean mass ± SD: 0.10 ± 0.06 mg, data from Versluijs et al. (2023)) to match the biomass of a single crane fly.

### 4.2 Body Size Reductions

Overall, our data suggest that over the past 20 years red knot chicks have faced an increasing demand‐resource mismatch with their main arthropod prey in their warming Arctic‐breeding grounds, causing both slower chick growth and smaller‐bodied birds after arrival in West Africa. Over the same period, the adult birds in Mauritania also appear to be shrinking, but at a slower rate (−0.02 phenotypic SDs vs. −0.04 in juveniles, Figure S6, Table S10). Shrinking body size can be expected to be slower in adults, which are slowly replaced by smaller‐bodied juveniles. At the same time, the diverging rates of body shrinkage between adults and juveniles show that these changes do not originate from measurement errors or changes in measurement methods. As changes in growth (measured in arctic Taimyr) as well as body size (measured in West Africa) are correlated with the timing of snowmelt (in Taimyr), and as variations in chick diet are correlated with body size, we have a strong case for body size reductions in juvenile red knots to originate from environmentally caused growth reductions (i.e., from developmental plasticity), rather than from natural selection.

While we observed juveniles in West Africa to have become smaller by 0.04% (for PC1, 0.03% for tarsus length) per day of earlier snowmelt, the reduction in chick size was 0.24% (for PC1, 0.35% for tarsus) for each day of earlier hatch. We were only able to collect biometric data on chicks up to fledging, yet chicks only reach final size after fledging yet before southward migration. However, we do not expect much compensation for reduced growth in this period (as they approach the asymptote of the growth curve then, Figure S3). It is more likely that the discrepancy between size reductions of chicks and full‐grown juveniles is caused by natural selection, which may dampen the effects of growth reductions if smaller‐sized juveniles have lower survival rates between fledging and arrival on the wintering grounds. This may be particularly true for wing length, which in our data from Mauritania is the measure with the lowest relative size reduction, possibly because selection against small wings already acted during their first migration from the high‐Arctic to West Africa. After arrival on the West‐African wintering grounds, red knots with shorter bills (which correlate with overall body size) appear to have a lower survival after arrival (van Gils et al. 2016). As a result, these selection pressures could lead to smaller‐sized red knots with relatively long bills and wings. This would coincidentally be in line with both Bergmann's and Allen's rule, that, respectively, relate smaller bodies and larger appendages to a warmer climate (Allen 1877; Bergmann 1847; McQueen et al. 2022), even though such changes may not provide climate‐related benefits (Nord et al. 2024). Since we see overall smaller bill and wing lengths in juveniles over time, selection against these smaller measures might contribute to the ongoing population decline in Mauritania (Oudman et al. 2020).

### 4.3 Developmental Plasticity and Body Size Changes—A General Pattern?

Body shrinkage caused by an increasing demand‐resource mismatch might be a general mechanism that could explain why in some species body size reductions are observed in synchrony with climate warming (Weeks et al. 2020; Youngflesh et al. 2022). Such causal chains remain rather unexplored. The chances of a demand‐resource mismatch likely differ between species (Zhemchuzhnikov et al. 2021) and this might therefore contribute to the observed variation in body size responses to climate warming (Gardner et al. 2011). Red knots may be especially sensitive due to extreme temperature changes in their breeding grounds, with temperatures increasing four times faster than elsewhere (Rantanen et al. 2022) in combination with little apparent response in the phenology of reproduction and migration. Long‐distance migration may increase the susceptibility of red knots to demand‐resource mismatches and corresponding body size reductions, as advancements in migratory timing may be restricted due to several potential limitations, including food conditions at wintering sites (Studds and Marra 2011), flexibility in departure timing (Conklin et al. 2013; Stanley et al. 2012) and potential for speeding up fuel deposition at staging sites along the migratory route (Lindström et al. 2019; Rakhimberdiev et al. 2018).

## Author Contributions

**Tim Oortwijn:** conceptualization, formal analysis, investigation, visualization, writing – original draft, writing – review and editing. **Thomas K. Lameris:** conceptualization, formal analysis, investigation, visualization, writing – original draft, writing – review and editing. **Mikhail K. Zhemchuzhnikov:** conceptualization, formal analysis, investigation, visualization. **Anne Dekinga:** investigation. **Job ten Horn:** investigation. **Dmitry Kutcherov:** investigation, writing – review and editing. **Simeon Lisovski:** investigation, writing – review and editing. **Theunis Piersma:** funding acquisition, investigation, writing – review and editing. **Eldar Rakhimberdiev:** formal analysis, writing – review and editing. **Mikhail Y. Soloviev:** investigation. **Bernard Spaans:** investigation. **Evgeny E. Syroechkovsky:** investigation. **Pavel S. Tomkovich:** investigation, writing – review and editing. **Elena Zhemchuzhnikova:** investigation. **Jan A. van Gils:** conceptualization, formal analysis, funding acquisition, investigation, supervision, visualization, writing – review and editing.

## Conflicts of Interest

The authors declare no conflicts of interest.

## Supporting information


Data S1.

## Data Availability

Data and R‐scripts are available in Dryad: https://doi.org/10.5061/dryad.3n5tb2rqb.
