# Supplementary material for: Demand‐Resource Mismatch Explains Body Shrinkage in a Migratory Shorebird
Source: Glob Chang Biol. 2025 Apr 16;31(4):e70170. doi: 10.1111/gcb.70170 (PMC12001007; doi:10.1111/gcb.70170)
Supplement: Supplementary file 1 — Data S1. [file GCB-31-e70170-s001.pdf]

Supplementary material to "Demand-resource mismatch explains body shrinkage in a migratory shorebird"

Juvenile and chick body size as percentage

To express juvenile body size as percentage, scaled body size values per individual were calculated, by also performing the principal component analysis including an imaginary individual of which its measures were 0. Subsequently, the absolute PC1-value of this '0-individual' (|-31.7|) was added to all individuals (resulting in values ranging from 29.3 to 34.2). Afterwards, all values were divided by the predicted size of a female hatched in 2003 (32.4), based on the best model, and multiplied by 100 (to be able to express the scaled values in percentages). To express size changes in phenotypic SDs, the slope of the effect was divided by the standard deviation of the residuals.

To express chick body size as percentage, relative to the size at the earliest relative hatch date (-1, or 1 day before the date of 50% snowmelt), the principal component analysis was also performed including an individual of which its measures were 0. Subsequently, the absolute PC1-value of this '0-individual' (|-8.8|) was added to all individuals (resulting in values ranging from 6.3 to 12.7). We fitted the best model of the effect of relative hatch date and average temperature on chick condition index also on these data, and subsequently scaled these data to the highest predicted chick condition index (at the earliest relative hatch date) expressed as a percentage.

Sex-specific growth models

We constructed sex-specific growth models for bill length, tarsus length and body mass in a similar fashion as described in the main text, but only using data for the years 2018 and 2019 as only in these years the chicks were sexed. For bill length and tarsus length we set sex-specific asymptote values as calculated from biometric values (see main text), while for body mass asymptote values were estimated in the model. We included a random effect of sex on parameters k and T (and A for body mass) and a random effect of bird identity on parameter k nested within year. The von Bertalanffy growth model performed best for bill length, the logistic model performed best for tarsus length and body mass (Table S11, Fig. S7). We compared model performance of these models with models on the same data where we did not include a random effect of sex and used non-sex-specific values for asymptotes. Models excluding sex always outperformed models where sex was included.

We further tested how sex-specific growth curves could affect our results of relative hatch date (RHD) on growth residuals by calculating the chick condition index (see main text) for bill length, tarsus length and body mass from the sex-specific growth models outlined above. These were then used in mixed effect models, where we included RHD, sex and their interaction as fixed effects and chick identity as random

intercept. We found that the best performing models never included the interaction effect of sex and RHD (Table S12), thus showing that not including sex in our models would not impact the effect of RHD we found.

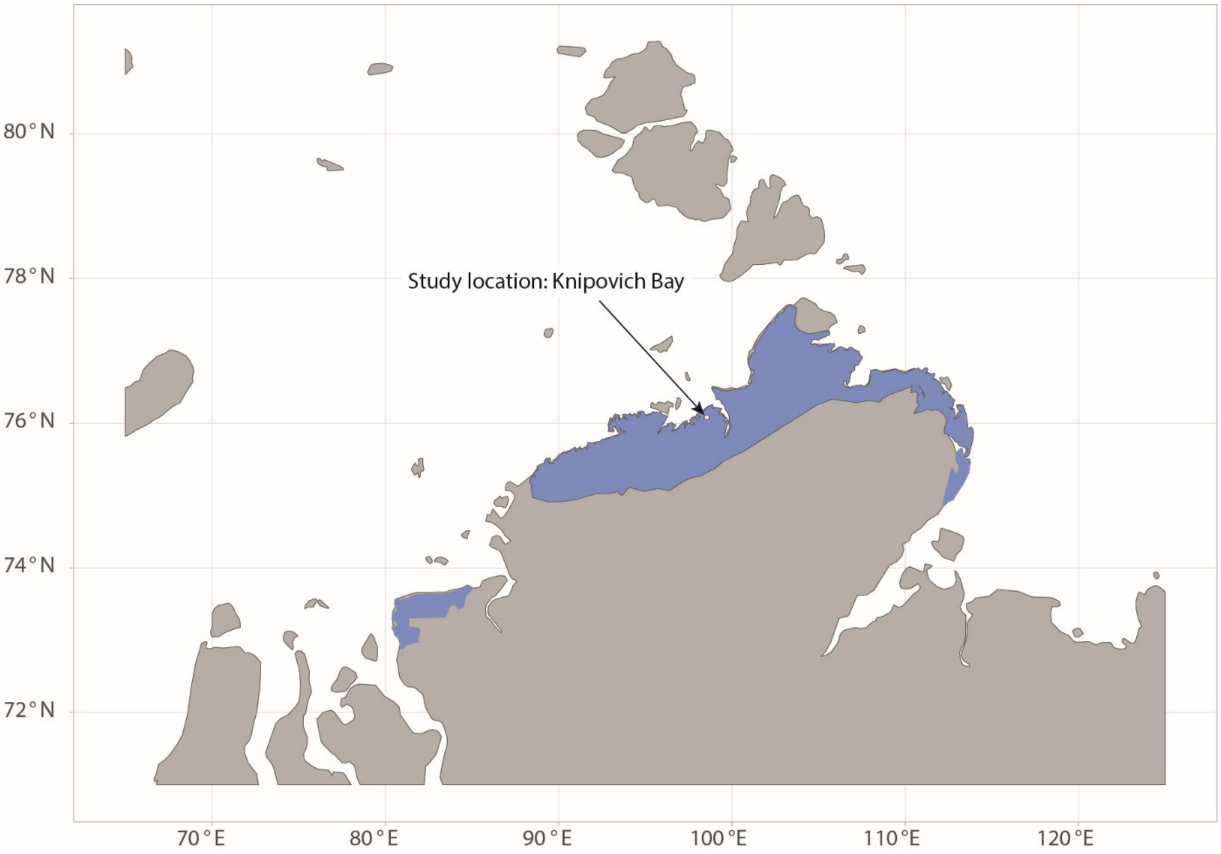

Figure S1. Breeding range (blue polygon) of red knot (*Calidris canutus canutus*), based on (Lappo et al., 2012), used to extract MODIS NDSI values.

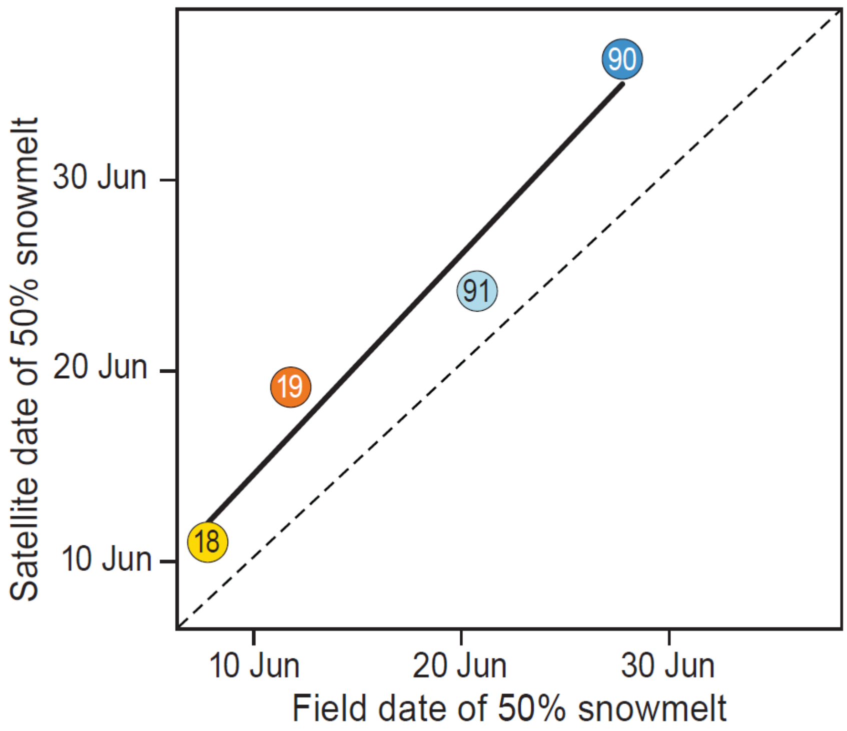

Figure S2. Date of 50% snowmelt as measured in the surroundings of the Arctic field site camp compared with the date of 50% snowmelt as measured from satellite imagery. The black line shows the linear correlation (Pearson's correlation coefficient = 0.97), the dashed line shows the y = x line. Numbers in dots indicate different years and colors correspond to those, as in Fig. 1 & S3.

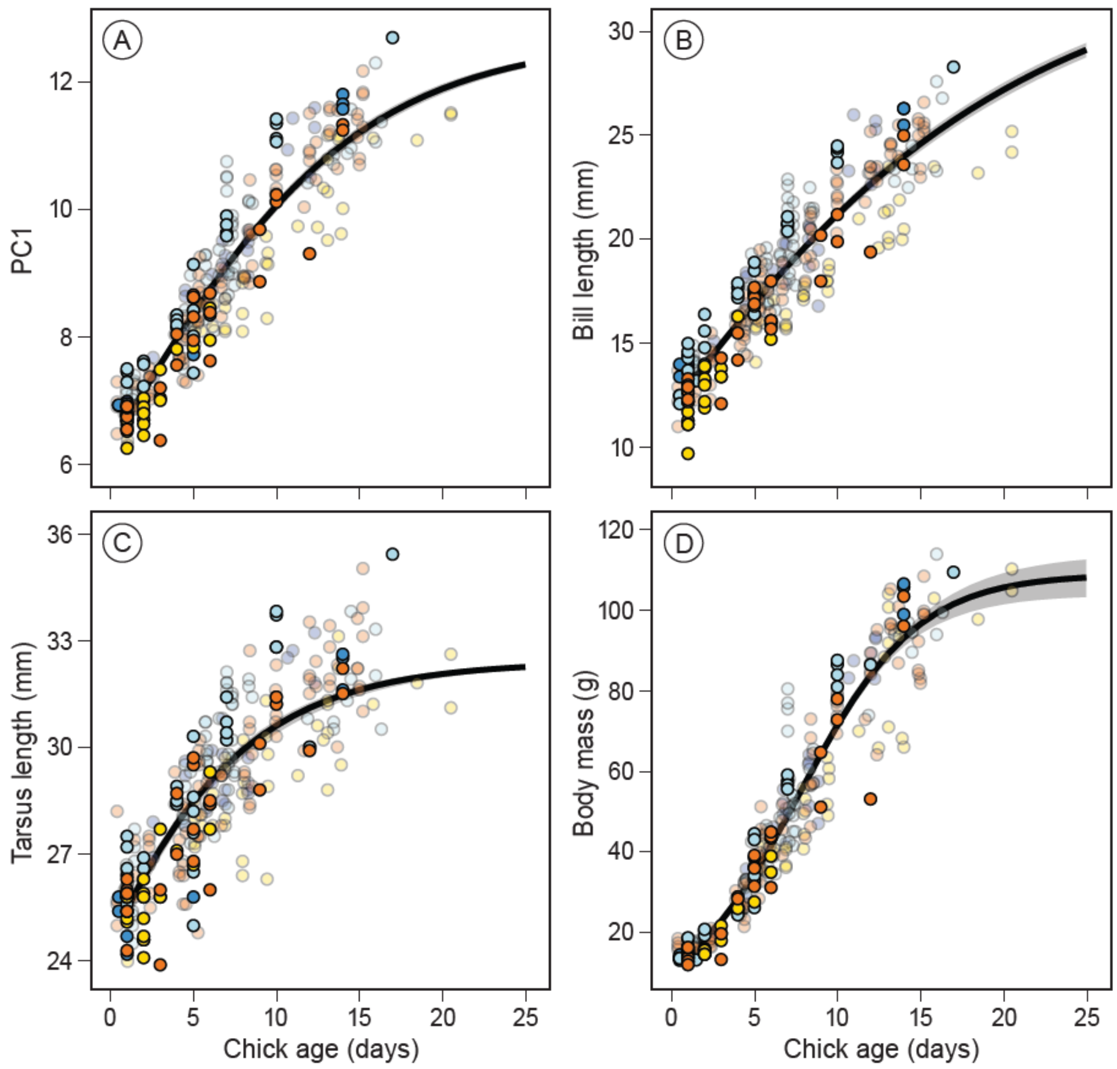

Figure S3. Growth of size and mass measurements of red knot chicks. Growth of (A) 1st principal component of bill and tarsus length (von Bertalanffy), (B) Bill length (von Bertalanffy), (C) tarsus length (logistic) and (D) body mass (logistic). Points show measurements of individual chicks with known (solid points) or predicted (transparent points) age. Colors indicate data from different years, with colors as in Fig. 1. Black lines show model predictions, grey areas show 95% Confidence Intervals.

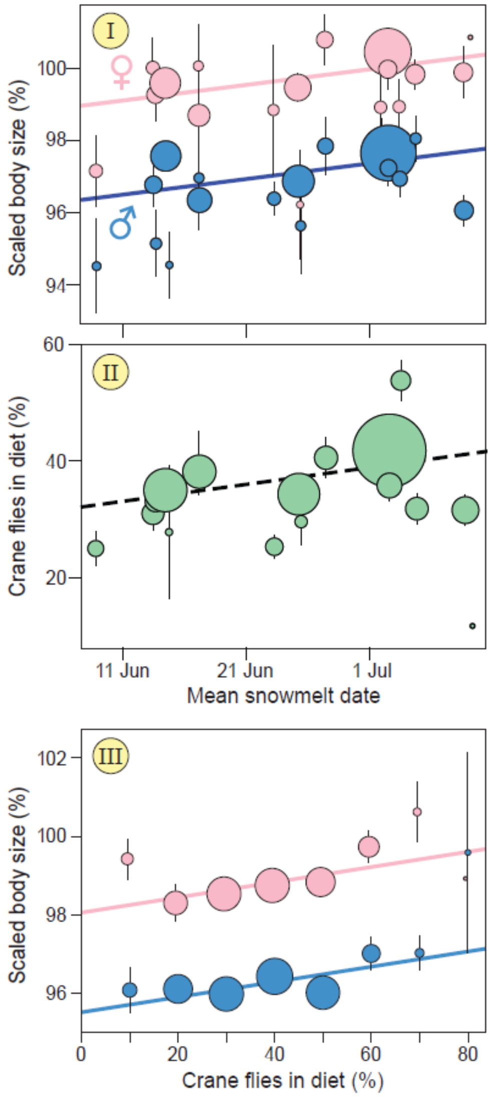

Figure S4. In years with later snowmelt, red knots consume proportionally more crane flies and are larger. (I) In years with later snowmelt, body sizes of juvenile red knots, caught on their wintering grounds in Mauritania, are larger. Dots are means ± se of the first principal component of tarsus length, bill length and wing length per year, scaled to a modeled female born on the 1st of July, dot size indicates sample size. **(II)** In years with later snowmelt, the proportion of crane flies in the diet of growing chicks is higher, which is estimated from stable isotopes in the feathers of juveniles (grown as chicks on the tundra) caught on the wintering grounds in Mauritania. Means ± se are shown per year and dot size indicates sample size. **(III)** Juveniles that have consumed proportionally more crane flies as chicks (method

explained in II) have larger bodies (method explained in I, here scaled to a modeled female on a 100 percent crane fly diet). Estimated crane fly proportions are rounded into 10%-bins.

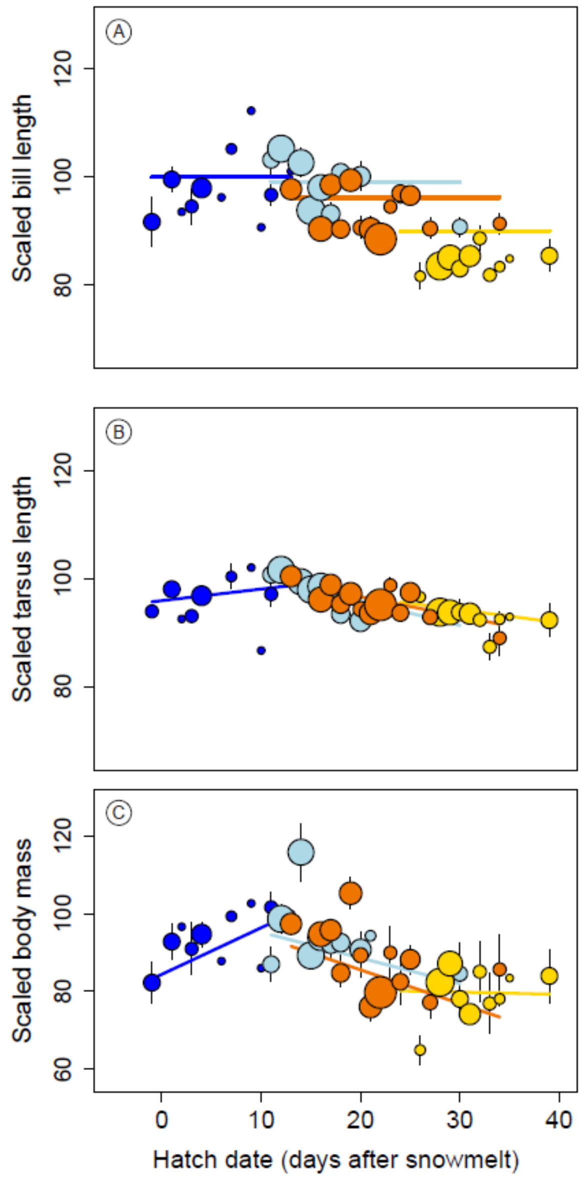

Figure S5. Relationships between chick condition indexes with relative hatch date. The relationship between relative hatch date (in days after snowmelt) and body size measures: (A) bill length, (B) tarsus length, and (C) body mass. Means ± se are shown per hatch date with dot size scaling with sample size (total 262 measurements from 208 chicks) and colors correspond to different years (see Fig. 1B). Condition indexes are scaled to percentages (%), with 100% set at the chick condition predicted at a relative hatch date of 0 in 1990 from GLMMs.

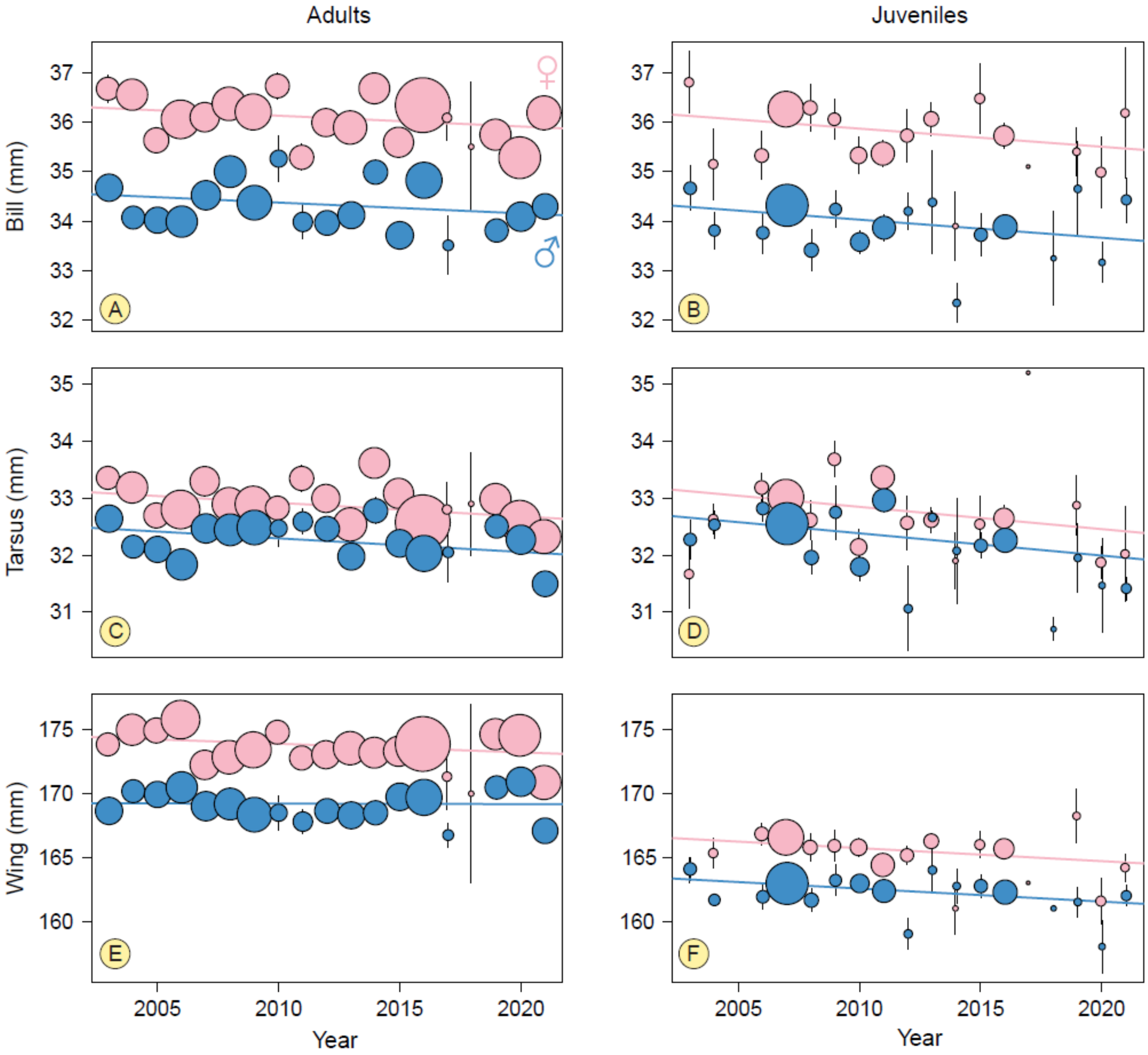

Figure S6. Declines in juvenile and adult body size over time. Size of bill, tarsus and wing length of adult (A, C and E, N=2165) and juvenile (D, E, F, N=592) red knots caught on wintering grounds in Mauritania between 2003 and 2021.

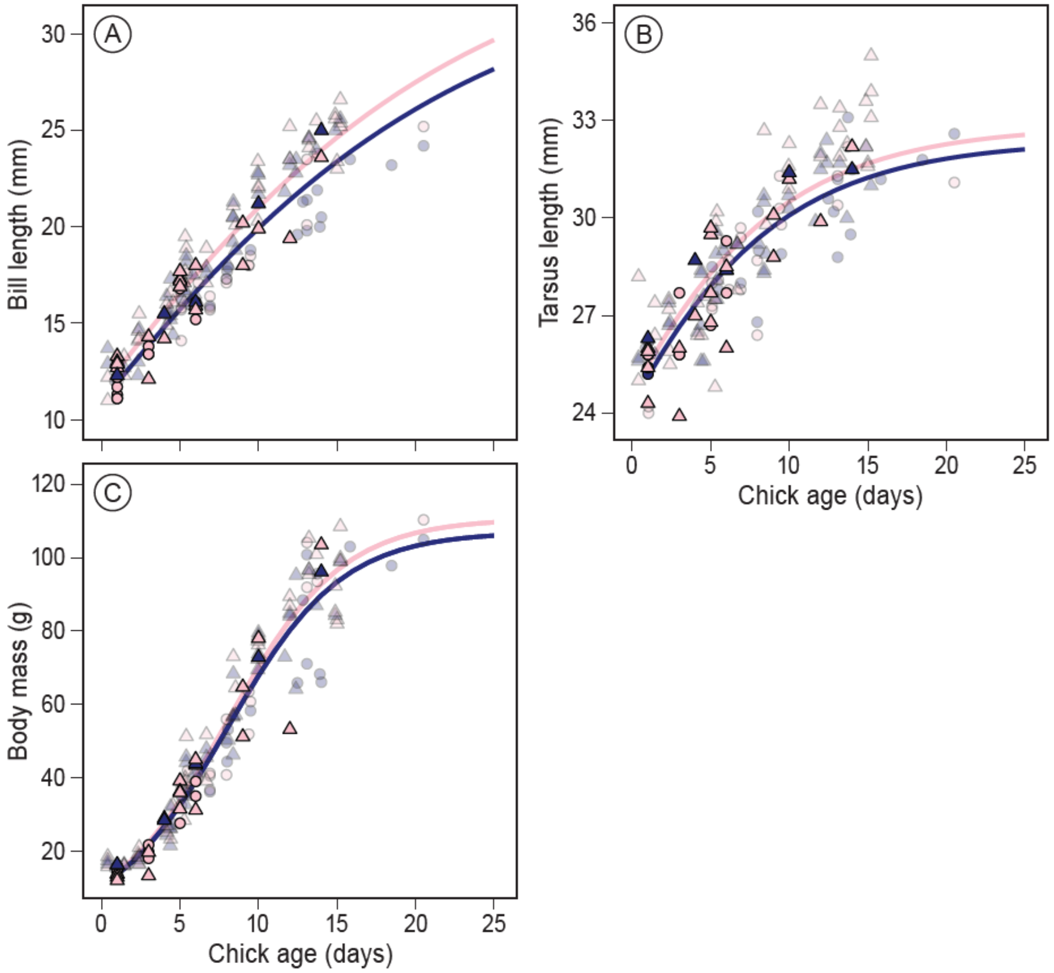

Figure S7. Growth of bill length, tarsus length and body mass over chick age, with points showing individual values and lines showing predictions from sex-specific growth models. Pink data points and lines show female values and predictions, blue data points and lines show male values and predictions. Data points from 2018 are depicted as dots, data points from 2019 are depicted as triangles.

Table S1. Total number of nests or broods found in each year using various methods. Numbers between brackets show additional nests found, but for which no chicks were measured (as nests were predated or chicks could not be located).

Year	flushed from / observed at nest	radio-tagged male	found with chicks	Total
1990	5		16	21
1991	8		21	29
2018	3 (1)	5 (2)	18	26
2019	2 (3)	1	21	24

Table S2. GLMs of snowmelt (SM) and hatch date (HD) over time (years, Y) and date of snowmelt. Hatch dates are analyzed in two sets of models, first including both observed hatch dates as well as hatch dates predicted from the 10th primary length of caught chicks (HD_{all}, see Methods), and second including observed hatch dates only (HD_{obs}). Models are ordered from low to high AICc, with the best model marked in bold. Model coefficients are reported separately for each model.

Model	Intercept	Slope (Y)	Degrees of freedom	AICc	delta AICc	Model weight
**SM ~ Y**	**1933.0**	**-0.87**	**3**	**140.1**	**0.0**	**0.824**
SM ~ 1	175.1		2	143.2	3.1	0.176
**HD_{all} ~ (Y)**	**193.2**		**3**	**544.5**	**0.0**	**0.968**
HD_{all} ~ Y + (Y)	233.8	-0.02	4	551.3	6.8	0.032
**HD_{obs} ~ (Y)**	**193.9**		**3**	**121.1**	**0.0**	**0.897**
HD_{obs} ~ Y + (Y)	409.0	-0.11	4	125.5	4.3	0.103
**HD_{all} ~ (Y)**	193.2		3	544.5	0.0	0.96
HD_{all} ~ SM + (Y)	193.5	-0.00	4	550.7	6.21	0.04
**HD_{obs} ~ (Y)**	193.9		3	121.1	0.0	0.88
HD_{obs} ~ SM + (Y)	168.9	0.14	4	125.1	3.98	0.12

Table S3. AICc values of von Bertalanffy, Gompertz and logistic growth models applied to data on bill length, tarsus length, body mass and their combined measure (PC1) as measured on growing Red Knot chicks. The best models are marked in bold.

	von Bertalanffy	Gompertz	logistic
PC1	**501.2**	505.0	542.1
Bill length	**1127.0**	1128.9	1139.6
Tarsus length	991.6	990.5	**987.4**
Body mass	2123.4	2113.1	**2102.2**

Table S4. Variables of growth models for all chicks in the dataset, including both known and predicted age, as well as for a subset including only chicks with known age. See methods for details how age prediction was conducted. Asymptotes (A) were fixed for bill length, tarsus length and PC1. Maximum growth rate (k) inflection point (t) and asymptote for body mass were calculated from the model via optimization.

		Both known and predicted age			Only known age		
	Growth model	Asymptote (A)	Growth rate (k)	Inflection point (t)	Asymptote (A)	Growth rate (k)	Inflexion point (t)
Bill length	von Bertalanffy	34.0	0.071	-1.660	34.0	0.079	-1.226
Tarsus length	logistic	32.4	0.171	-6.622	32.4	0.175	-6.259
Body mass	logistic	109.0	0.282	7.726	116.2	0.292	7.911
PC1	von Bertalanffy	6.9	0.139	4.707	6.9	0.160	4.654

Table S5. Ivlev indexes and stable nitrogen isotope ratios of most consumed arthropod families. Ivlev index is calculated from the proportion of arthropod families found in the diet and the proportion found in the pitfall traps (see Methods). N is the number of individuals measured for stable nitrogen isotope ratios, except for Chironomidae, where N is the number of measurements (with multiple individuals per measurement because of their small size, same for one Staphylinidae sample).

Family	Ivlev index	$\delta^{15}N$		
		Mean	Standard deviation	Sample size

Anthomyiidae	-0.61 ± 0.06	6.98	0.75	9
Chironomidae	0.23 ± 0.08	4.37	0.99	9
Chrysomelidae	-0.71 ± 0.04	4.59	1.30	10
Empididae	-0.79 ± 0.04	7.06	1.32	10
Muscidae	-0.90 ± 0.02	7.12	1.11	10
Staphylinidae	-0.81 ± 0.05	6.37	0.75	10
Tipulidae	0.11 ± 0.06	2.77	1.69	10

Table S6. Estimates of unstandardized path coefficients λ (including mean and standard deviation) and their probabilities P(|b|>0) from the structural equations model. Significant parameters are marked in **bold**. Note that these coefficient values have not been backscaled (as opposed to the values reported in Fig. S1).

Variable	Effect of	on	mean	SD	P(|b|>0)
**b1.2**	**Time**	**Snowmelt**	-0.484	0.214	**0.99**
**b1.3**	**Time**	**Crane fly proportion in diet**	-0.345	0.099	**1.00**
**b1.4**	**Time**	**Body size**	-0.087	0.044	**0.98**
b2.2	Snowmelt	intercept	0.092	0.299	0.63
b2.3	Snowmelt	Crane fly proportion in diet	-0.073	0.109	0.76
**b2.4**	**Snowmelt**	**Body size**	0.075	0.043	**0.96**
b3.3	Crane fly proportion in diet	intercept	-0.043	0.102	0.67
**b3.4**	**Crane fly proportion in diet**	**Body size**	0.064	0.037	**0.96**
**b4.4**	**Body size**	**intercept**	0.554	0.051	**1.00**
**b4.s**	**Sex**	**Body size**	-1.031	0.070	**1.00**

Table S7. GLMs of body size (PC1), bill length (BL), tarsus length (TL), wing length (WL) and proportion crane flies in the diet (CF) measured in juvenile birds in Mauritania as affected by sex (S) and hatch year (HY) included as a continuous variable. In CF-models, HY is included as random variable. Models are ordered from low to high AICc, with the most parsimonious model marked in bold.

Model	Degrees of freedom	AICc	Δ-AICc	weight
Body size (PC1)				
**PC1 ~ S + HY**	**4**	**1803.1**	**0**	**0.73**
PC1 ~ S * HY	5	1805.1	1.98	0.27
PC1 ~ S	3	1819.4	16.31	0
PC1 ~ HY	3	1989.8	186.72	0

PC1 ~ 1	2	1997.9	194.78	0
Bill length (BL)				
**BL ~ S + HY**	**4**	**2181.5**	**0**	**0.66**
BL ~ S * HY	5	2183.4	1.89	0.25
BL ~ S	3	2185.5	3.96	0.09
BL ~ HY	3	2363	181.45	0
BL ~ 1	2	2363.6	182.03	0
Tarsus length (TL)				
**TL ~ S + HY**	**4**	**1887.8**	**0**	**0.71**
TL ~ S * HY	5	1889.6	1.8	0.29
TL ~ S	3	1896.9	9.15	0.01
TL ~ HY	3	1907.9	20.13	0
TL ~ 1	2	1915.5	27.75	0
Wing length (WL)				
**WL ~ S + HY**	**4**	**3040.7**	**0**	**0.69**
WL ~ S * HY	5	3042.3	1.67	0.3
WL ~ S	3	3049.5	8.85	0.01
WL ~ HY	3	3172.7	132.06	0
WL ~ 1	2	3177.1	136.43	0
Crane fly proportion in diet (CF)				
CF ~ S + HY + (HY)	5	4698.9	0	0.53
**CF ~ HY + (HY)**	**4**	**4700.2**	**1.31**	**0.28**
CF ~ S * HY + (HY)	6	4701.1	2.04	0.19
CF ~ S + (HY)	4	4711.3	12.32	0
CF ~ 1 + (HY)	3	4712.8	13.87	0

Table S8. GLMMs of the chick condition index for 1st principal component, bill length, tarsus length and body mass. Fixed effects included relative hatch date (RHD), the average temperature during the 3 days before measurement of the chick (AT) and year as factor (Y). A random intercept of chick identity (ID) is included. Models are ordered from low to high AICc, with the most parsimonious model marked in bold. Models within 2 ΔAICc are marked in italics. Model coefficients are reported separately for each model.

	Intercept	AT	RHD	Degrees of freedom	AICc	ΔAICc	weight
First principal component							
PCI ~ RHD + AT + Y + (ID)	*-0.01*	*0.02*	*-0.01*	*8*	*-122.0*	*0.00*	*0.46*

**PCI ~ RHD + AT + (ID)**	**0.01**	**0.02**	**-0.01**	**5**	**-121.6**	**0.42**	**0.38**
PCI ~ RHD + AT + Y + RHD*Y + (ID)	-0.08	0.02	0.00	11	-119.3	2.71	0.12
PCI ~ AT + Y + (ID)	-0.07	0.02		7	-117.3	4.67	0.05
PCI ~ RHD + Y + (ID)	0.12		-0.01	7	-100.9	21.02	0.00
PCI ~ AT + (ID)	-0.21	0.03		4	-97.8	24.21	0.00
PCI ~ Y + (ID)	0.07			6	-90.4	31.59	0.00
PCI ~ RHD + (ID)	0.25		-0.01	4	-80.6	41.35	0.00
PCI ~ (ID)	-0.01			3	-27.5	94.50	0.00
Bill length							
*BCI~ RHD + AT + Y + RHD*Y + (ID)*	*-0.03*	*0.01*	*0.01*	*11*	*-758.3*	*0.00*	*0.45*
**BCI ~ AT + Y + (ID)**	**-0.01**	**0.01**		**7**	**-757.8**	**0.48**	**0.35**
BCI ~ RHD + AT + Y + (ID)	*0.00*	*0.01*	*0.00*	*8*	*-756.7*	*1.68*	*0.20*
BCI ~ RHD + AT + (ID)	0.01	0.01	0.00	5	-742.7	15.61	0.00
BCI ~ RHD + Y + (ID)	0.05		0.00	7	-729.3	29.07	0.00
BCI ~ Y + (ID)	0.04			6	-726.7	31.60	0.00
BCI ~ AT + (ID)	-0.07	0.01		4	-707.7	50.61	0.00
BCI ~ RHD + (ID)	0.09		0.00	4	-692.7	65.60	0.00
BCI ~ (ID)	0.00			3	-626.3	132.05	0.00
Tarsus length							
**TCI ~ RHD + AT + Y + RHD*Y + (ID)**	**-0.02**	**0.00**	**0.00**	**11**	**-949.5**	**0.00**	**0.78**
TCI ~ RHD + AT + Y + (ID)	0.01	0.00	0.00	8	-946.3	3.17	0.16
TCI ~ RHD + Y + (ID)	0.02		0.00	7	-943.7	5.82	0.04
TCI ~ RHD + AT + (ID)	0.01	0.00	0.00	5	-941.7	7.84	0.02
TCI ~ AT + Y + (ID)	-0.01	0.00		7	-931.0	18.48	0.00
TCI ~ RHD + (ID)	0.04		0.00	4	-930.5	19.04	0.00
TCI ~ AT + (ID)	-0.03	0.00		4	-926.2	23.29	0.00
TCI ~ Y + (ID)	0.00			6	-924.2	25.27	0.00
TCI ~ (ID)	0.00			3	-898.4	51.09	0.00
Body mass							
**MCI ~ RHD + AT + Y + RHD*Y + (ID)**	**-0.06**	**0.01**	**0.01**	**11**	**-327.3**	**0.00**	**0.63**
MCI ~ RHD + AT + (ID)	0.01	0.01	0.00	5	-324.3	2.96	0.14
MCI ~ RHD + AT + Y + (ID)	0.01	0.01	0.00	8	-323.8	3.50	0.11
MCI ~ AT + Y + (ID)	-0.02	0.01		7	-322.9	4.39	0.07
MCI ~ RHD + Y + (ID)	0.05		-0.01	7	-321.7	5.57	0.04
MCI ~ Y + (ID)	0.03			6	-318.7	8.61	0.01
MCI ~ AT + (ID)	-0.08	0.01		4	-315.6	11.71	0.00
MCI ~ RHD + (ID)	0.10		-0.01	4	-314.5	12.73	0.00
MCI ~ (ID)	-0.01			3	-292.1	35.13	0.00

Table S9. GLMMs of the fraction of crane flies in the diet of chicks as determined from stable isotopes in growing feathers. Fixed effects included relative hatch date (RHD), year (Y) and their interaction. A random intercept of brood identity (B) and year is included. Models are ordered from low to high AICc, with the most parsimonious model marked in bold. Models within 2 ΔAICc are marked in italics.

Model	Degrees of freedom	AICc	Δ-AICc	weight
CF ~ RHD + (B) + (Y)	*5*	*259.3*	*0.00*	*0.36*
CF ~ RHD + Y + (B) + (Y)	*6*	*259.4*	*0.19*	*0.33*
**CF ~ (B) + (Y)**	**4**	**260.7**	**1.47**	**0.17**
CF ~ RHD + Y + RHD*Y + (B) + (Y)	7	262.5	3.23	0.07
CF ~ Y + (B) + (Y)	5	262.8	3.59	0.06

Table S10. Yearly trends in red knot body size (both expressed as PC1 value as well as in phenotypic standard deviations), tarsus, bill and wing length (all expressed in mm) over the period 2003 – 2021 from captures in the wintering grounds in Mauritania. Values are from the most parsimonious models out the comparison of GLMs, that explained size changes by year, sex and their interaction. Values are given for juvenile and adult birds, as well as separate values for female and male adult birds when the best explaining model included an interaction between year and sex.

	Juvenile	Adult	Adult F	Adult M
Body size (PC1)	-0.04		-0.03	-0.01
Body size (phenotypic SDs)	-0.04		-0.03	-0.01
Tarsus (mm)	-0.04	-0.02		
Bill (mm)	-0.04	-0.02		
Wing (mm)	-0.10		-0.07	-0.004

Table S11. AICc values of growth models including random effects of sex, year nested in sex and chick identity nested in year.

	von Bertalanffy	Gompertz	logistic
Body mass (incl. sex)	1095.0	1086.9	**1078.1**
Body mass	1089.0	1082.1	**1073.8**
Bill length (incl. sex)	**563.4**	564.1	570.0
Bill length	**550.4**	550.9	556.1
Tarsus length (incl. sex)	514.2	513.2	**510.3**
Tarsus length	514.1	513.2	**510.3**

Table S12. GLMMs of chick condition for PC1, bill length (BL) and tarsus length (TL) as explained by relative hatch date (RHD), sex (S) and their interaction (RHD*S), and chick identity included as a random intercept (ID). Models are ordered from smallest to highest ΔAIC. The best performing models are marked in bold**.**

Model	Intercept	β relative hatch date	Degrees of freedom	AICc	Δ AICc	Model weight
Body mass (BM)						
**BM ~ RHD + (ID)**	**0.166**	**-0.007**	**4**	**-197.50**	**0.00**	**0.67**
BM ~ RHD + S + (ID)	0.170	-0.007	5	-195.40	2.08	0.24
BM ~ RHD + S + RHD*S + (ID)	0.178	-0.008	6	-193.30	4.24	0.08
BM ~ (ID)	-0.009		3	-188.70	8.83	0.01
BM ~ S + (ID)	-0.008		4	-186.60	10.95	0.00
Bill length (BL)						
**BL ~ RHD + S + (ID)**	**0.153**	**-0.007**	**5**	**-363.50**	**0.00**	**0.59**

BL ~ RHD + S + RHD*S + (ID)	0.132	-0.006	6	-362.30	1.22	0.32
BL ~ RHD + (ID)	0.170	-0.007	4	-359.80	3.73	0.09
BL ~ S + (ID)	-0.018		4	-332.10	31.39	0.00
BL ~ (ID)	-0.003		3	-328.90	34.61	0.00
Tarsus length (TL)						
**TL ~ RHD + (ID)**	**0.073**	**-0.003**	**4**	**-503.20**	**0.00**	**0.57**
TL ~ RHD + S + RHD*S + (ID)	0.090	-0.004	6	-501.40	1.83	0.23
TL ~ RHD + S + (ID)	0.071	-0.003	5	-501.30	1.99	0.21
TL ~ (ID)	-0.004		3	-484.90	18.34	0.00
TL ~ S + (ID)	-0.006		4	-483.20	20.07	0.00
